# The classification of *Crithidia luciliae* immunofluorescence test (CLIFT) using a novel automated system

**DOI:** 10.1186/ar4510

**Published:** 2014-03-14

**Authors:** Francesca Buzzulini, Amelia Rigon, Paolo Soda, Leonardo Onofri, Maria Infantino, Luisa Arcarese, Giulio Iannello, Antonella Afeltra

**Affiliations:** 1 Clinical Medicine and Rheumatology, Campus Bio-Medico University of Rome, via Alvaro del Portillo 21, 00128 Rome, Italy; 2 Computer Science & Bioinformatics Laboratory, Campus Bio-Medico University of Rome, via Alvaro del Portillo 21, 00128 Rome, Italy; 3 Immunology and Allergology Laboratory Unit, S. Giovanni di Dio Hospital, Via Torregalli 3, 50143 Florence, Italy

## Abstract

**Introduction:**

In recent years, there has been an increased demand for computer-aided diagnosis (CAD) tools to support clinicians in the field of indirect immunofluorescence. To this aim, academic and industrial research is focusing on detecting antinuclear, anti-neutrophil, and anti-double-stranded (anti-dsDNA) antibodies. Within this framework, we present a CAD system for automatic analysis of dsDNA antibody images using a multi-step classification approach. The final classification of a well is based on the classification of all its images, and each image is classified on the basis of the labeling of its cells.

**Methods:**

We populated a database of 342 images—74 positive (21.6%) and 268 negative (78.4%)— belonging to 63 consecutive sera: 15 positive (23.8%) and 48 negative (76.2%). We assessed system performance by using k-fold cross-validation. Furthermore, we successfully validated the recognition system on 83 consecutive sera, collected by using different equipment in a referral center, counting 279 images: 92 positive (33.0%) and 187 negative (67.0%).

**Results:**

With respect to well classification, the system correctly classified 98.4% of wells (62 out of 63). Integrating information from multiple images of the same wells recovers the possible misclassifications that occurred at the previous steps (cell and image classification). This system, validated in a clinical routine fashion, provides recognition accuracy equal to 100%.

**Conclusion:**

The data obtained show that automation is a viable alternative for *Crithidia luciliae* immunofluorescence test analysis.

## Introduction

Anti-double-stranded DNA (anti-dsDNA) antibodies are serological markers of systemic lupus erythematosus (SLE), considered to be markers of disease activity and organ damage. They entered to be part of classification criteria for SLE, according to the recommendation of the American College of Rheumatology and they have been confirmed as immunological criteria for SLE in the recently published SLICC (Systemic Lupus International Collaborating Clinics) criteria [1,2]. Several assays are now available for the detection of dsDNA autoantibodies. Currently used techniques in clinical laboratories vary from the Crithidia luciliae immunofluorescence test (CLIFT) to radioimmunoassays (RIAs) (Farr assay and PEG assay) or easily automatized enzyme-linked immunosorbent assays (ELISAs) [3,4]. In the CLIFT, the antigen source is the kinetoplast of the hemoflagellate *Crithidia luciliae*, which contains naked circular DNA. The test detects medium- to high-avidity isotype-specific anti-dsDNA antibodies, thus coupling high disease specificity (98% to 100%) with good sensitivity (47% to 55%) [5]. With respect to the technique, indirect immunofluorescence (IIF) is affected by several issues limiting test reliability and reproducibility [6]. Therefore, in recent years, there has been an increase in demand for computer-aided diagnosis (CAD) tools offering support both to clinicians and to diagnosticians. Indeed, CAD systems may be useful in many ways: (a) they can be adopted as a second reader, thus augmenting the clinician’s capabilities and reducing errors; (b) they allow physicians to perform a pre-selection of the cases to be examined, enabling them to focus attention on only the most relevant cases; and (c) they can be used as a tool for training and education of specialized medical personnel. Since the validation of the use of digital images for diagnostic purpose in the field of indirect immunofluorescence [6], there has been research and industrial interest in developing CAD systems with applications in the fields of antinuclear antibodies (ANAs), anti-neutrophil antibodies, and CLIFT detection [7-24].

We recently presented an experimental model of the CAD system for automated ANA detection on HEp2 cells (SLIM-system), which provides a reliable identification of negative samples and a flexibility that permits this application to be used for different purpose [24]. The aforementioned research efforts prove that there is a growing interest in developing reliable and useful automatic CAD systems in IIF. In this respect, we present a CAD system for automatic analysis of dsDNA images, whose performance has been validated in clinical routine fashion to have achieving an accuracy equal to 100%.

### System architecture

The system collects several images of the same well since these images do not cover the entire well surface at the used microscope magnification. This feature permitted us to exploit a certain degree of redundancy, integrating information extracted from different images of the same well. The proposed CAD system applies a multi-step classification approach, so that final classification of a well is based on the classification of all its images. Furthermore, each image is classified on the basis of the classification of its cells.

In the first classification step, we worked on the cells. To this aim, we first detected presumed kinetoplast by applying a threshold-based classification. In this way, we were able to detect the compact set of pixels more fluorescent than other parts that are candidates to be a kinetoplast. Conversely, the absence of such regions permitted us to label the image as negative. Next, we considered only those images containing at least one candidate kinetoplast region. In such images, we located the cells and extracted from them a set of features. The feature set permitted us to divide the cells with a candidate kinetoplast in those containing and not containing a true kinetoplast. This set was composed of measures belonging to intensity histogram, Fourier transform (FT), circular local binary pattern (LBP), and morphological descriptors. Features computed over the intensity histogram were related to the number of high fluorescent pixels and to the absolute maximum intensity value. Furthermore, features extracted from the grey-level co-occurrence matrix describe the image texture, which varies between cells where only the kinetoplast is fluorescent and cells where the basal body, the nucleus, or artifacts are fluorescent. Features computed from the FT catch information related to spatial frequency in the image. For instance, the greater the number of fluorescent objects inside a cell, the higher the frequency in the spectrum. Circular LBP features describe image texture with reference to circular information. Finally, the morphological descriptors catch information on shape and intensity of presumed kinetoplast. Given this set of features, we applied a supervised classifier, namely a Support Vector Machine, which assigns a positive or a negative label to each cell on the basis of the knowledge incorporated during the training phase of the method.

In the second step of the proposed multi-step approach, we classified the images: given the set of labels assigned to the cells, each image is classified by majority voting over its cells. This combination rule, selected among other criteria after preliminary tests, assigns to the input image the label corresponding to the class receiving the majority of votes [25].

The third and last step classifies the well on the basis of the labels associated with its images. To this aim, we again used the majority voting rule, assigning to the well the label of the class with the majority number of images. Furthermore, the system suspends the decision when an equal number of images have opposite labels. This choice corresponds to a conservative criterion that aims at minimizing the misclassification risk.

## Methods

We initially populated a database of annotates images by using slides of *Crithidia luciliae* (CL) substrate (The Binding Site) at the fixed dilution of 1:10 as recommended by guidelines [26]. Two specialists took five CL images per well, on average, with an acquisition unit consisting of the fluorescence microscope (Orthoplan; Leitz, Stuttgart, Germany) coupled with a 50-W mercury vapor lamp and with a digital camera (F145C; Allied Vision Technologies, Stadtroda, Germany). Images have a resolution of 1,388 × 1,038 pixels and a color depth of 24 bits and are stored in a bitmap format. We used two different magnifications (25- and 50-fold) to test robustness to cell size variation. The images then were blindly classified by two experts of IIF, who were asked to reach consensus on the cases about which they disagreed.

This image data set consists of 342 images—74 positive (21.6%) and 268 negative (78.4%)—belonging to 63 sera: 15 positive (23.8%) and 48 negative (76.2%). One hundred fifty-four images have been acquired by using 25-fold magnification, and the remaining 188 by using the 50-fold magnification.

Moreover, specialists labeled a set of cells belonging to images with fluorescent cells since our recognition approach requires the labels of individual cells to train the corresponding classifier. This procedure was carried out at a workstation monitor since at the fluorescence microscope it is not possible to observe one cell at a time. Notice that the use of digital images in IIF for diagnostic purposes has been discussed [6]. At the end, the cells’ data set consisted of 1,487 cells belonging to 34 wells: 928 labelled as positive (62.4%) and 559 as negative (37.6%). This means that, on average, each image contained approximately eight cells.

These sets of cells and well images were used to develop and test the proposed recognition approach. In keeping with common practice in the pattern recognition and machine learning fields, we assessed system performance by using the k-fold cross-validation. To avoid any bias introduced by this procedure, we divided the set of 1,487 cells into several subsets, one for each well, and then performed a one-well-out cross-validation, in which the cells of one well constitute the test set and the others the training set.

Furthermore, we validated the recognition system in a daily routine fashion. In this respect, we used 83 consecutive sera of outpatients and inpatients of the Campus Bio-Medico, University Hospital of Rome. These images were acquired in two different rounds. In the first round, we collected 48 sera by using a 50-fold magnification lens and the aforementioned equipment and substrate. In the second round, other 35 consecutive sera were acquired using slides of CL substrate (Inova Diagnostics, Inc., San Diego, CA, US). We used the fluorescence microscope Eurostar II coupled with a led and with a digital camera (DX40; Kappa, Gleichen, Germany). In this case, images have a resolution of 1,392 × 1,040 pixels and a color depth of 24 bits and are stored in jpeg format. The images were acquired by using the 40-fold magnification. At the end, this validation set consisted of 83 wells, resulting in a total of 279 images. This means that in this phase we acquired an average of three images per well. The distributions of wells in the positive and negative classes were 35.0% and 65.0% (29 and 54 wells), respectively. In the validation phase, we collected a total of 279 images: 92 positive (33.0%) and 187 negative (67.0%).

The next section will present the results we achieved: the performance of the system has been estimated by the accuracy, the specificity, the sensitivity, and the precision. To provide a deep insight in the data, we also reported the contingency table.

## Results

### Cell classification

Cell classification performance has been estimated on the 1,487 cells described in section Materials and methods. Percentages of recognition accuracy on cell images acquired at 25- and 50-fold are 94.4% and 94.0%, respectively. Sensitivity and specificity on cell images acquired at 25-fold are 94.7% and 93.8%, respectively, whereas on cell images acquired at 50-fold, they are 97.1% and 89.9%, respectively. These similar values suggest that cell classifier is robust to cell size variation.

### Image classification

As presented in section Materials and methods, image classification consists of two steps. In the first one, we apply a threshold-based classification which aims at detecting clearly negative images. The system labeled 221 images as negative and 121 images as positive. All positive images passed this phase, whereas 17.5% of negative images, in this step, were misclassified (Table 1). Such errors are expected since threshold-based classification looks for fluorescent connected regions, corresponding to presumed kinetoplast. However, this step does not permit a satisfactory performance in well classification, since the discrimination between true- and false-positive samples remains an open issue.

**Table 1:** **Contingency table of threshold**-**based image classification**

	**Hypothesized class**	
	**Positive**	**Negative**
True class		
Positive	74 (100.0%)	0 (0.0%)
Negative	47 (17.5%)	221 (82.5%)
Total	121	221

The second step applies cell classifier to recognize whether the image is positive (that is, it contains true fluorescent kinetoplasts). The system now works on single-cell classification to recognize whether the images having candidate kinetoplast regions contain the true fluorescent kinetoplast. Of the 121 images labelled as positive by the previous step, 73 images (98.6%) were classified as true positive and 46 images (97.9%) as true negative, as shown in the contingency table illustrated in Table 2. Only two images have been misclassified. Integrating the results of threshold-based and the cell-based classification, we found that most of the false-positive images given by threshold-based classification are now correctly classified, with only one false positive (0.4%).

**Table 2:** Contingency table of overall image classification

	**Hypothesized class**	
	**Positive**	**Negative**
True class		
Positive	73 (98.6%)	1 (1.4%)
Negative	1 (0.4%)	267 (99.6%)

Finally, we analyzed whether magnification affects the recognition performance. Classification accuracies on images acquired by using 25- and 50-fold magnifications are 99.4% and 99.5%, respectively. When the images are acquired by using 25-fold magnification, sensitivity and specificity are 96.2% and 100.0%, respectively. When the images are acquired by using 50-fold magnification, sensitivity and specificity are 100.0% and 99.3%, respectively. These results confirm that the system is robust to variation of magnifications.

### Well classification

The integration of information from multiple images of the same wells enables us to recover the possible misclassifications that occurred at the previous steps. As described in Table 3, the system correctly classified 98.4% of wells (62 out of 63) whereas only one has been rejected since it consists of two images labeled to the two opposite classes. Indeed, the system suspended the decision and asks the physician for the final decision.

**Table 3:** Performance of cell, image, and whole well classification

	**Accuracy**	**Sensitivity**	**Specificity**	**Precision**
	**%**	**%**	**%**	**%**
Cell classification	94.2	95.5	92.1	95.3
Image classification	99.4	98.6	99.6	98.6
Well classification	98.4	93.3	100	100
System validation	100	100	100	100

### System validation

CAD systems can be a valuable tool since they may offer support to physicians in different working scenarios. However, it is normal to have some skepticism about the true usefulness of CAD systems in daily practice. In particular, physicians are often doubtful of performance of such systems because their application in clinical practice requires a careful assessment in a daily routine fashion. In this respect, we verified the strength of our CAD system on 83 consecutive sera, as reported in section Materials and methods. Each well has been classified according to the approach described so far: the system first labels its images and then takes the final decision. The last row of Table 3 shows that the proposed CAD system correctly classified all samples.

## Discussion

The proposed CAD system for automatic analysis of CLIFT applies a multi-step classification approach, as illustrated in section System architecture. The first step identifies the clearly negative images, applying a threshold-based classification. Images supposed to be positive are subsequently classified by a classification system that discriminates true- from false-positive images by using different features (for example, morphological descriptors that provide information on shape and intensity of presumed kinetoplast). This classification system is able to correctly recognize all false-positive images given by the threshold-based classification, with only one false positive remaining (0.4%). Hence, the multi-step approach is one of the strengths of the proposed system since it permits recovery of the possible misclassifications that occur in each step.

To provide the final result, the CAD system performs well classification, integrating information from multiple images. On this phase, the system correctly classified 98.4% of wells, whereas for one well it suspends the classification and asks the physician for the final decision. This happens since there are two images for such a well that are labelled to the two opposite classes (positive and negative).

Furthermore, the system went through a validation phase run on clinical routine, where it achieves an accuracy of 100%. It is worth noting that images of sera used in this validation phase were collected in two different rounds by using substrates produced by different companies, different microscopes, different lighting sources, different digital cameras, and two different compression algorithms, as described in section Materials and methods. Despite this large variability, the results show that the system is robust and is able to adapt to different working scenarios. Indeed, the classification algorithm employed in our proposal automatically tunes its free parameters to the characteristics of the images at hand. We therefore deem that our methodology for automated CLIFT standardization can be adapted to many laboratories, regardless of which equipment is used.

The standardized detection of anti-dsDNA antibodies is a topic that has attracted recent research interest [3,4,15-17]. Roggenbuck and colleagues [3] compared Farr assay, ELISA, and CLIFT, remarking that CLIFT can be automated, thus reducing the interlaboratory variability similar to HEp-2 IIF in antinuclear antibody detection [10,12]. Elsewhere, Roggenbuck and colleagues [4] suggested that the Aklides (Medipan, Berlin, Germany) reading system can also be used for the automated reading of images given by the modified CLIFT presented in [27]. In another report, the authors [16] reviewed and summarized the general and specific features of seven recent available commercial systems for automation of the IIF method, pointing out that the Aklides (Medipan) and the Image Navigator (Immuno Concepts, Sacramento, CA, USA) systems are able to detect antibodies to dsDNA. While for the Image Navigator system no data are available in the literature [15], the results of the Aklides systems in case of automated CLIFT analysis are reported in [16,17]. In these papers the authors collected 44 sera and then they compared the Aklides diagnostic performance on dsDNA samples with the traditional visual interpretation made by laboratory experts. The results showed that the Aklides system got values of accuracy, sensitivity, specificity and precision equal to 90.9%, 90.9%, 90.9% and 76.9%, respectively. Furthermore, in the case of critical samples, the Aklides system does not permit the clinicians to work in parallel with the automated system, according to what was reported in the articles.

## Conclusions

The need of automatic applications in indirect immunofluorescence for autoimmunity comes from the high inter- and intra-laboratory variability, which is due to subjective image evaluation and to the different level of expertise between operators while they read and interpret the images. In addition, immunologists and clinical pathologists have to face the increasing laboratory workload observed in recent years. CAD tools are able to respond to this demand, but currently a critical issue is the development and validation of systems providing high accuracy and also giving the clinician the possibility to operate on critical samples.

Returning to our proposal, we deem that the global architecture adopted in our CAD system provides the following benefits:

•the threshold-based approach reduces the occurrence of false-negative classification, whereas the cell-based classification permits clinicians to distinguish between true- and false-positive images;

•the initial threshold-based classification allows a rapid categorization of several images;

•the system is tolerant with respect to misclassification in cell recognition; in fact, if enough cells per image are available, it is reasonable that misclassified cells, if limited, do not affect image classification;

•the proposed system, validated in a clinical routine fashion, provides recognition accuracy equal to 100%, showing that automation is a viable alternative for CLIFT analysis.

The classification system has proved to be able to adapt to the different substrates and equipment. Nevertheless, in future works, we plan to extend our validation tests to substrates produced by other companies to further assess the robustness and adaptability of the classification system. Furthermore, we plan to investigate how the CAD works with diluted sera to introduce the chance to differentiate between low and high positive.

## Abbreviations

ANA: antinuclear antibody; anti-dsDNA: anti-double-stranded DNA (antibody); CAD: computer-aided diagnosis; CL: *Crithidia luciliae*; CLIFT: *Crithidia luciliae* immunofluorescence test; dsDNA: double-stranded DNA; ELISA: enzyme-linked immunosorbent assay; FT: Fourier transform; IIF: indirect immunofluorescence; LBP: circular local binary pattern; RIA: radioimmunoassay; SLE: systemic lupus erythematosus.

## Competing interests

The authors declare that they have no competing interests.

## Authors’ contributions

FB performed image acquisition and classification and drafted the manuscript. AR participated in the design of the study, performed image acquisition and classification, and drafted the manuscript. PS participated in the conception, design, and development of software tools for data analysis and interpretation and drafted the manuscript. LO participated in the design, development, and test of software tools for data analysis and interpretation and revised the manuscript. MI performed image acquisition during the validation phase and revised the manuscript. LA carried out the immunofluorescence tests and revised the manuscript. GI participated in the design of the study, supported data analysis, and revised the manuscript. AA participated in the design of the study and revised the manuscript. All the authors read and approved the final manuscript.
